# Reciprocal Associations Between Sleep, Mental Strain, and Training Load in Junior Endurance Athletes and the Role of Poor Subjective Sleep Quality

**DOI:** 10.3389/fpsyg.2020.545581

**Published:** 2020-10-09

**Authors:** Maria Hrozanova, Christian A. Klöckner, Øyvind Sandbakk, Ståle Pallesen, Frode Moen

**Affiliations:** ^1^Department of Neuromedicine and Movement Science, Faculty of Medicine and Health Sciences, Norwegian University of Science and Technology, Trondheim, Norway; ^2^Department of Psychology, Faculty of Social and Educational Sciences, Norwegian University of Science and Technology, Trondheim, Norway; ^3^Department of Psychosocial Science, Faculty of Psychology, University of Bergen, Bergen, Norway; ^4^Norwegian Competence Center for Sleep Disorders, Haukeland University Hospital, Bergen, Norway; ^5^Optentia, The Vaal Triangle Campus of the North-West University, Vanderbijlpark, South Africa; ^6^Department of Education and Lifelong Learning, Faculty of Social and Educational Sciences, Norwegian University of Science and Technology, Trondheim, Norway

**Keywords:** sleep, athlete, mental strain, training load, sleep quality

## Abstract

The importance of adequate sleep for athletic functioning is well established. Still, the literature shows that many athletes report sleep of suboptimal quality or quantity. To date, no research has investigated how bidirectional variations in mental and physiological states influence sleep patterns. The present study, therefore, investigates reciprocal associations between sleep, mental strain, and training load by utilizing a prospective, observational design. In all, 56 junior endurance athletes were followed over 61 consecutive days. Unobtrusive, objective measurements of sleep with novel radar technology were obtained, and subjective daily reports of mental strain and training load were collected. The role of subjective sleep quality was investigated to identify whether the reciprocal associations between sleep, mental strain, and training load depended on being a good versus poor sleeper. Multilevel modeling with Bayesian estimation was used to investigate the relationships. The results show that increases in mental strain are associated with decreased total sleep time (TST, 95% CI = −0.12 to −0.03), light sleep (95% CI = −0.08 to −0.00), and sleep efficiency (95% CI = −0.95 to −0.09). Further, both mental strain and training load are associated with subsequent deceased rapid eye movement (REM, respectively, 95% CI = −0.05 to −0.00 and 95% CI = −0.06 to −0.00) sleep. Increases in TST, light, deep, and REM sleep are all associated with subsequent decreased training load (respectively, 95% CI = −0.09 to −0.03; 95% CI = −0.10 to −0.01; 95% CI = −0.22 to −0.02; 95% CI = −0.18 to −0.03). Finally, among poor sleepers, increases in sleep onset latency are associated with increases in subsequent mental strain (95% CI = 0.09–0.46), and increases in deep sleep are associated with decreases in subsequent training load (95% CI = −67.65 to 11.43). These results offer novel insight into the bidirectional associations between sleep, mental strain, and training load in athletes and demonstrate the detrimental effects of mental strain on sleep, likely caused by mental activation incompatible with sleep. An increased need for recovery, suggested by increased TST and time in different sleep stages, is associated with subsequent self-regulatory reduction of training loads by the athletes. In poor sleepers, increases in deep sleep may suggest an elevated need for physiological recovery.

## Introduction

Optimal sleep is recognized for its positive contributions to physical performance and psychological well-being in the athletic population (Thun et al., 2015; Kroshus et al., 2019). During sleep, learning and memory processes consolidate and strengthen retrieval of factual information and facilitate procedural motor skills, which contribute to athletic performance (Walker and Stickgold, 2004). Sleep is also important for energy reestablishment (Jung et al., 2011); emotional regulation (Palmer and Alfano, 2017); and the functioning of immune, cardiovascular, endocrine, and metabolic systems (Manzar et al., 2015). In athletic populations, sleep has, consequently, been recognized as an essential aspect of recovery (O’Donnell et al., 2018), enabling athletic progression and performance improvement (Kellmann et al., 2018).

Although it seems paramount that athletes obtain optimal sleep, existing evidence suggests that this is not the case for many athletes. For example, one study based on actigraphic recordings finds that athletes have poorer sleep than non-athletes in terms of sleep latency, sleep efficiency, and sleep fragmentation (Leeder et al., 2012), and another study finds poorer subjective sleep quality among athletes (Bender et al., 2018). Poor sleep in athletes may reflect sport-specific demands, such as challenging organizational, competitive (Hanton et al., 2005), and psychophysiological stressors (Nixdorf et al., 2015; Campbell et al., 2018). Indeed, previous research has established that sleep of athletes may be negatively influenced by increases in training load (Kolling et al., 2016) and early morning scheduling of training sessions (Sargent et al., 2014) and matches (Juliff et al., 2015) as well as psychological aspects (e.g., worry) of the stress response (Hrozanova et al., 2019).

Still, the mechanisms causing sleep disruptions in athletes have not been thoroughly investigated. Both physical, i.e., training load, as well as mental components of stress, i.e., mental strain, may represent sleep-impairing factors. Importantly, bidirectional associations between physical and mental stressors and sleep may also be present. Regarding the effects of training load and sleep, some studies find no significant associations (Knufinke et al., 2018; O’Donnell et al., 2019; Lastella et al., 2020), and others find that increased training load leads to reductions in sleep duration (Kolling et al., 2016) and sleep efficiency (Teng et al., 2011). Research investigating the effects of sleep on training load has found that self-determined training load is lower in partially sleep deprived elite athletes than in teammates who obtained adequate sleep (Cook et al., 2012). In another study, sleep restricted to 4 h for three nights led to performance impairments, slower response time, and loss in joint coordination (Mah et al., 2019). Overall, studies on the bidirectional associations between training load and sleep are few and present contrasting results.

Likewise, only a handful of studies explore the mentally straining effects of athletic participation on sleep. During competition, sleep onset latency is found to increase, and sleep quality deteriorates, likely due to stress-related cognitive activity associated with competing (Lastella et al., 2014; Juliff et al., 2015). One large cross-sectional study identifies perceived stress as the most important predictor of poor sleep quality (Hrozanova et al., 2019). Regarding the effects of sleep on mental functioning in athletes, another cross-sectional study finds associations between poor sleep quality and confusion, depression, and fatigue (Andrade et al., 2019). However, research based on more suitable, longitudinal designs is lacking.

It is likely that the ambiguous results regarding the association between sleep, training load, and mental strain in athletic populations may be attributed to methodological challenges with sleep monitoring. Most studies to date use subjective sleep assessment due to the low cost and availability of such measurements. However, subjective measures may be subject to recall (Coughlin, 1990), common method (Podsakoff et al., 2003), and social desirability (Grimm, 2010) biases. Several existing studies also utilize objective measures of sleep, typically employing actigraphy or, to a lesser extent, polysomnography (PSG). The former has generally limited specificity (Marino et al., 2013) and cannot differentiate between sleep stages, whereas PSG suffers from practical drawbacks related to cost, skills, and time, limiting its feasibility in long-term sleep assessment (Pallesen et al., 2018).

As a solution to the methodological limitations in previous research, the present study performed objective, repeated measurements of sleep by employing novel technology in junior endurance athletes. The novel sleep monitoring device is based on an impulse radio ultra-wideband (IR-UWB) radar sensor technique and machine learning and has been validated against PSG with substantial agreement on both sleep/wake and sleep staging (Toften et al., 2020). The primary aim of the present study is to investigate the reciprocal and temporal associations between sleep, mental strain, and training load. The secondary aim is to investigate whether being a good versus poor sleeper has moderating effects on the associations between sleep, mental strain, and training load. The latter aim is important for identifying athletes vulnerable to the effects of fluctuations in stress and sleep. Ecological validity is ensured by collecting data during a dynamic 2-month period representative of a typical preparatory phase of the season, documenting real-life fluctuations in sleep, training load, and mental strain as they occur without any interventions or artificial constraints.

## Materials and Methods

### Participants

Participants were recruited from high schools specialized for winter endurance sports in Norway. In these schools, training is a part of athletes’ educational plan, and they also train after school in their respective sports teams. From the schools, a total of 80 students were invited to information meetings about the research project. Athletes who showed interest were asked to sign up for the study by contacting the researchers. Those interested then signed and returned an informed consent form and were, thus, included. Due to equipment constraints (sleep monitors) the maximum number of participants that could be included was 60. Of these 60 initially included, four dropped out: three were excluded due to lack of willingness to commit, and one participant did not provide a reason. Thus, 56 (93.3% of 60) athletes completed the study, of which 37 were male and 19 female. The mean age of the sample was 17.6 (range 17–19) years. In all, 40 athletes practiced cross-country skiing, and 16 athletes practiced biathlon.

### Ethical Considerations

All athletes gave their informed consent to participate. According to the Norwegian Health Research Act (§17.1), the lower legal age for providing consent to participate in health-related research is 16 years. Hence, because all participants in this study were 16 years or older, parental consent was not necessary. The Regional Committee for Medical and Health Research Ethics (REC) in Central Norway approved the study (project ID 2017/2072/REK Central Norway).

### Procedure

A prospective observational cohort study design was employed with daily monitoring of sleep, mental strain, and training load. In the period of data collection, no interventions were implemented. Training load was self-regulated with the aim of improving performance and followed up by professional coaches. Mental strain was self-assessed based on the athletes’ own perception and understanding of strain. Athletes were instructed to follow their normal sleep and wake patterns and were informed that monitoring was meant to assess their normal, daily functioning, representative of the preparatory phase of the season.

Before the assessment was initiated, participants completed a questionnaire assessing basic demographics and the Pittsburgh Sleep Quality Index (PSQI, Buysse et al., 1989) on the web-based survey service Questback. The PSQI was administered in the beginning of the study as this was logistically most feasible. After questionnaire completion, equipment for monitoring of sleep (i.e., Somnofy sleep monitor), mental strain (i.e., well-being questionnaire, WQ), and training load (i.e., training diaries) was handed out along with instructions for use. Athletes were instructed to complete the WQ each evening before bedtime and to complete training diaries every day upon completion of their training sessions to eliminate issues with past recall and inaccurate data entries.

### Instruments

#### Somnofy Sleep Monitor

The Somnofy sleep monitor is a novel, fully unobtrusive tool for sleep assessment based on an IR-UWB pulse radar and Doppler technology. It is recently shown to be an adequate measure of sleep and wake as well as sleep stages in a healthy adult population. When validated against the “gold standard” of sleep measurement, PSG, epoch-by-epoch analyses of the Somnofy sleep monitor showed that accuracy of measurements determined by Cohen’s kappa was 0.97 for sleep, 0.72 for wake, 0.75 for light sleep, 0.74 for deep sleep, and 0.78 for REM sleep. Therefore, some divergence between the Somnofy and PSG exists, which should be taken into consideration when interpreting its output. Still, Somnofy seems to provide more accurate sleep staging than several comparable non-obtrusive sleep assessment alternatives (Peake et al., 2018). For a full technical overview of the sleep monitor, including its limitations and results of its validation, see Toften et al., 2020.

The following sleep variables, derived from the sleep monitor, are included in the analyses: sleep onset latency; total sleep time; time in light, deep, and REM sleep; and sleep efficiency. These sleep variables are described in greater detail in Table 1.

**TABLE 1:** Descriptions of the measured sleep variables collected with the Somnofy sleep monitor in 56 junior endurance athletes.

Sleep variable	Abbreviation	Description
Sleep onset latency	SOL	Time from lights off to sleep onset
Total sleep time	TST	Total sleep time achieved during the night
Light sleep	–	Time in the light stages of sleep (stage N1 and N2)
Deep sleep	–	Time in the deep stages of sleep (stage N3)
Rapid eye movement sleep	REM	Time in REM sleep (stage R)
Sleep efficiency	SE	The ratio of time from lights off to leaving bed

*For more information on sleep stages, see American Academy of Sleep Medicine (2020).*

#### App-Based Well-Being Questionnaire

The WQ, a self-report stress scale based on recommendations for monitoring of overtraining in athletes (Hooper and Mackinnon, 1995), was used for daily assessment of mental strain. The WQ is utilized in multiple studies investigating wellness and fatigue in athletes (McLean et al., 2010; Buchheit et al., 2013a,b; Gallo et al., 2016; Conte et al., 2018; Lukonaitiene et al., 2020) and is routinely used in various athletic populations due to its low cost, user-friendly design, and ease of implementation.

The original WQ includes questions about fatigue, sleep quality, muscle soreness, stress levels, and mood rated on a visual analog scale. We slightly adapted the original WQ to fit the purpose of the current study. The question about stress levels was reframed as a question about worry and rumination, and the question about mood was kept the same. This was done in order to obtain markers of both cognitive (worry/rumination) and affective (mood) components of the stress response. Athletes were asked to provide daily scores on the WQ with visual rating scales, ranging from 0 to 10 for each question, where “0” indicates low levels of stress, and “10” indicates high levels of stress. For the purposes of the present study, only data regarding the two questions about worry/rumination and mood are analyzed. The scores of the two questions were added and then averaged to obtain a measure of mental strain with both emotional and cognitive components. The WQ was built into the smartphone-based Somnofy app, to which all participants had access.

##### Training diaries

Participants recorded their daily training sessions in digital diaries. The platforms used included the Norwegian Olympic Federation training diary^1^, the Bestr training diary^2^, or a custom Excel training diary provided by the Norwegian Ski Federation. The type of training recorded in these diaries was the same, independent of diary, and the information extracted from athletes’ training diaries was used to calculate training load. For that purpose, endurance and strength as well as plyometric and speed training were included. Other forms of training, such as mobility, stretching, and biathletes’ shooting practice were excluded.

For endurance training, athletes self-reported the time spent in different aerobic intensity zones in line with a five-zone intensity scale developed by the Norwegian Top Sport Centre (Olympiatoppen). The scale is frequently used in endurance sports in Norway, and junior athletes are systematically taught to independently self-report time spent in these different intensity zones as a part of their education in the high schools specialized for elite sports. When self-reporting, athletes do not use the heart rate and blood lactate information (provided as reference values for the five different zones) and rely on their perception of intensity as this is regularly calibrated toward these physiological measures. Previously, this self-report method was validated against heart rate and blood lactate levels and showed that endurance athletes self-report their training data accurately as both training duration and intensity distribution closely match the heart rate and lactate data (Sylta et al., 2014).

However, the 5-zone intensity scale is not clearly anchored in underlying physiological events (Boulay et al., 1997). In order to match the physiological parameters with the zones, we recalculated the 5-zone scale into a 3-zone scale. In the 3-zone scale, the zones correspond to the first and second lactate turning points (Boulay et al., 1997; Seiler and Kjerland, 2006). The 3-zone scale differentiates between low-intensity training (LIT), which encompasses all training below the first lactate threshold [zones 1 and 2; <2 mM blood lactate, 60–81% of maximal heart rate (HR_max_)]; moderate-intensity training (MIT), corresponding to the intensity between the first and second lactate threshold (zone 3; 2–4 mM blood lactate, 82–87% of HR_max_); and high-intensity training (HIT), corresponding to intensity above the second lactate threshold (zones 4 and 5; >4 mM blood lactate, >87% of HR_max_) (Seiler and Kjerland, 2006). Therefore, from the original scale reported, we merged zones 1 and 2 into LIT, zone 3 represented MIT, and zones 4 and 5 were merged into HIT. This approach has been successfully applied in previous studies (e.g., Solli et al., 2017).

Based on the training intensities reported (LIT, MIT, HIT), training impulse (TRIMP) scores were calculated in order to quantify training load (Pyne and Martin, 2011). Endurance TRIMP scores were calculated by multiplying the total duration (min) in each endurance intensity zone by a constant for each intensity zone (1 for LIT, 2 for MIT, 3 for HIT). TRIMP scores for strength, plyometric, and speed training were calculated by multiplying the total duration (min) in this training mode by a constant of 1.5. See Table 2 for the breakdown of the 5-zone intensity scale with typical heart rate and blood lactate values and subsequent utilization of the 3-zone scale anchored in the first and second lactate turning point. Also, see Table 2 for the TRIMP weights, how reference information on the zones corresponds to the different session ratings of perceived exertion (Foster, 1998), and examples of typical training sessions for each category (Seiler and Kjerland, 2006).

**TABLE 2:** The intensity scale used to determine the training load of endurance and strength, plyometric, and speed training in this study.

5-zone Norwegian Olympic Federation’s Intensity Scale			Physiologically accurate 3-zone scale			Reference information	
			
Zone	Lactate (mmol/L)	Heart rate (% of max.)	Lactate turning point	Intensity zone	TRIMP weight	sRPE	Typical training sessions
**Endurance training**							
1	0.8–1.5	55–72	< 1^st^ lactate threshold	Low-intensity training	1	0–4	Warm-up/cool down, > 90 min
2	1.5–2.5	72–81			Moderate duration, 45–90 min
3	2.5–4.0	82–87	1^st^–2^nd^ lactate threshold	Moderate-intensity training	2	5–6	Continuous sessions, 30–60 min; intervals with 6–15 min periods
4	4.0–6.0	88–92	> 2^nd^ lactate threshold	High-intensity training	3	7–10	Competitions, intervals with 4–8 min periods Competitions, intervals with 1–5 min periods
5	6.0–10.0	92–97	
**Strength, plyometric, and speed training**							
–	–	–	–	–	1.5	–	Plyometric/strength exercises, 4–30 reps; speed training, 5–15 s periods

*sRPE, session rating of perceived exertion. The lactate and heart rate values refer to typical values for the training in the different intensity zones, although these parameters were not directly measured in this study. The training impulse (TRIMP) weight for strength, plyometric, and speed training is based on the assessment of load by researchers and elite coaches in cross-country skiing.*

The resulting TRIMP scores were then employed as markers of total training load (TTL), the primary measure of training load in this study. TTL was calculated by adding the accumulated endurance TRIMP scores with the strength, plyometric, and speed training TRIMP scores for each training day (e.g., Solli et al., 2017, 2019). In case athletes trained more than once per day, all scores for one particular day were summed, resulting in one score per day.

#### Pittsburgh Sleep Quality Index

The PSQI (Buysse et al., 1989) is a self-report measure of subjective sleep quality. The index, adapted into Norwegian by Pallesen et al. (2005), includes 19 questions that investigate factors associated with sleep quality over the past month. In all, 4 questions had open entry answers, and the rest were scored on a 4-point (0–3) Likert scale. The responses were then added to obtain a global composite score, ranging from 0 to 21. Low scores signify good sleep quality. A cutoff of 5 was used to categorize respondents into good sleepers (≤5) and poor sleepers (>5). The Norwegian version of the PSQI has been shown to have good psychometric properties (Pallesen et al., 2005).

### Data Collection Compliance

**FIGURE 1:**
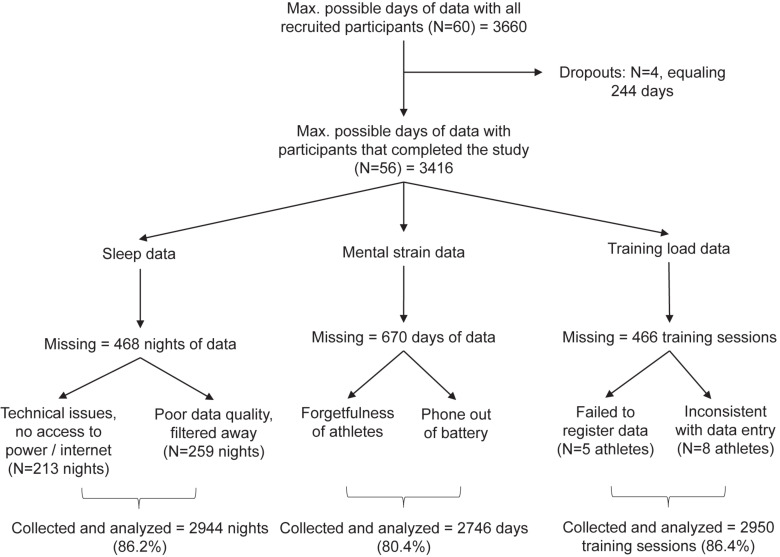
A breakdown of data-collection compliance with reasons for missing data. Percentages were calculated out of 3416, the maximum number of observations in each of the daily measured variables with 56 participants.

Due to the demanding day-to-day nature of the data collection, technical issues and challenges with participants’ inconsistent reporting were present, leading to missing data. Figure 1 presents a breakdown of collected and missing data with reasons for the lack of compliance.

### Statistical Analyses

The collected data created a nested data structure, in which up to 61 repeated measurements were nested within 56 individual athletes. In order to allow for dependence among the responses and to avoid excessive Type I errors and biased parameter estimates, multilevel modeling in Mplus, version 8.3 (Muthén and Muthén, 2017), was utilized to carry out the statistical analyses. The use of Mplus allowed for the investigation of both within (repeated measurement units, level 1) and between level (individual athletes, level 2) relations between sleep, mental strain, and training load.

The analyses were carried out in two temporal directions. First, we investigated associations between mental strain and training load at time 1 and subsequent sleep at time 1. In these analyses, the mental strain and training load reported on a particular day (e.g., Monday) were tested for associations with sleep that started on the evening of the same day that mental strain and training load were reported (e.g., sleep onset on Monday evening). In these analyses, mental strain and training load, thus, were the independent variables, and the different sleep variables represented the dependent variables. Second, we investigated associations between sleep and next-day (subsequent) mental strain and training load. In these analyses, sleep that started on a particular evening (e.g., Monday night) was tested for associations with mental strain and training load experienced the following day (e.g., Tuesday). In these analyses, the different sleep variables comprised the independent variables, and next-day mental strain and training load represented the dependent variables.

**FIGURE 2:**
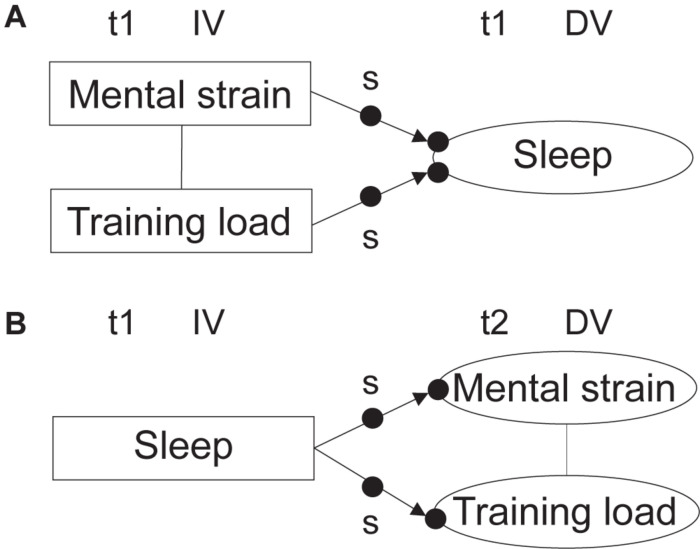
A visual representation of the primary aims in the current study, with investigation of the effects of mental strain and training load on sleep **(A)**, and of sleep on next day mental strain and next day training load **(B)**. Filled, unmarked circles represent random intercepts. Filled circles marked ‘s’ represent random slopes. The figure specifies temporal relationships between the variables, as well as dependent and independent variables.

**FIGURE 3:**
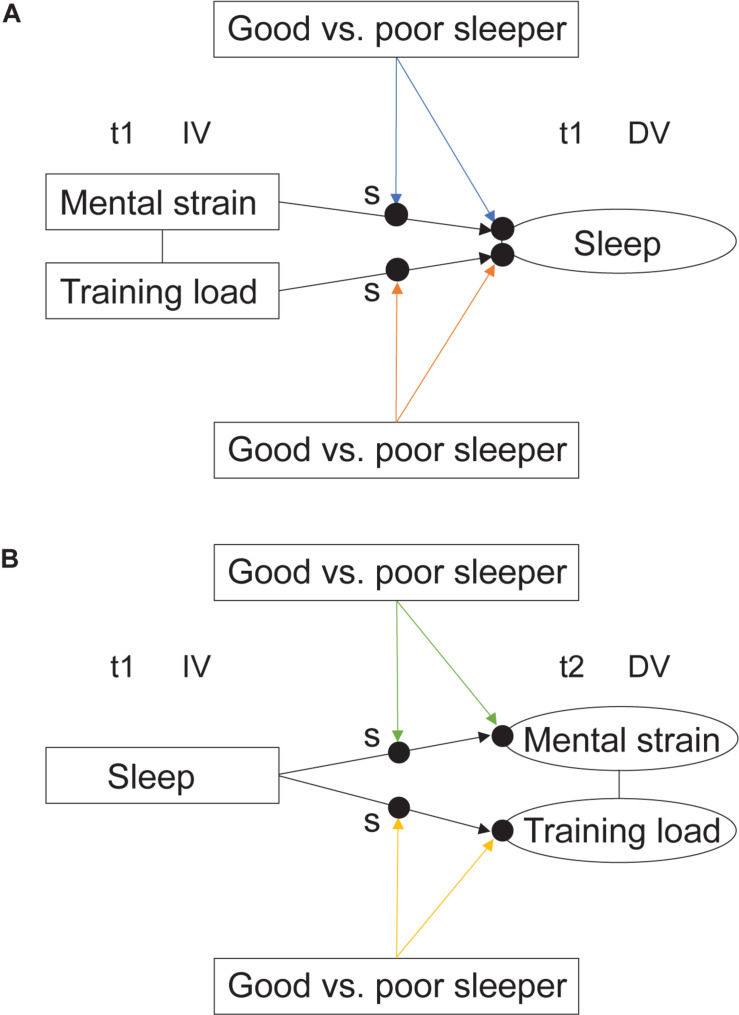
A visual representation of the secondary aim in the current study, with moderating effects of being a good vs. a poor sleeper on **(A)** the effect of mental strain on sleep (blue arrows), and the effect of training load on sleep (orange arrows), and **(B)** the effect of sleep on next day mental strain (green arrows), and the effect of sleep on next day training load (yellow arrows). Filled, unmarked circles on the response variables represent the random intercepts, while filled circles marked with ‘s’ represent the random slopes. The figure specifies temporal relationships between the variables, as well as dependent and independent variables.

Two-level random intercept and random slope models, which assume that variation between individuals is at their intercept (random intercept) and that the effects of the explanatory variables vary between individuals (random slope), were used to investigate the research questions in both aim 1 (Figure 2) and aim 2 (Figure 3). For aim 1 analyses, training load scores were divided by 100 to scale down the values in order to communicate the effects more clearly. Bayesian estimation was utilized in all random slope models investigated in this study as the models were computationally too complex for maximum likelihood estimation. In Bayesian analysis, prior distributions for parameters are combined with data likelihood to form posterior distributions for parameter estimates. Bayesian estimation is based on posterior probability distributions and uses the credibility interval for significance testing (Muthén, 2010). Grand mean centering of predictor variables was implemented to reduce multicollinearity and to establish a meaningful zero point: the intercept became an average across the whole sample. On the between level, the results show the estimated means and variances of the predictor variables across athletes with average values on the measured outcome parameters. For all multilevel models, *R*^2^-values, stating the explained variance were reported. *P*-values were set at < 0.05 for all models. For readers interested in the specific effect of sport (cross-country skiing and biathlon) in the models assessing reciprocal associations between sleep, mental strain, and training load, results are available in Supplementary Materials.

## Results

### Descriptive Statistics

Descriptive statistics for the sleep parameters, mental strain, training load, and subjective sleep quality are reported in Table 3.

**TABLE 3:** Descriptive statistics for objectively measured sleep patterns, mental strain, training load, and subjective sleep quality, reporting means and SD, in 56 junior endurance athletes.

		*M*	*SD*
Objectively measured	SOL (h)	00:35	00:31
sleep patterns	TST (h)	07:33	01:12
	Light (h/%)	04:16/56.3	00:52/7.2
	Deep (h/%)	01:24/18.7	00:23/5.1
	REM (h/%)	01:53/24.7	00:31/5.2
	SE (%)	79.0	10.2
Mental strain	(au)	3.5	1.6
Training load	Training sessions pp (N)	52.3	23.1
	Training missed due to resting pp (N)	9.4	6.0
	Training missed due to sickness pp (N)	6.6	5.8
	TTL (TRIMP)	103.8	83.8
Subjective sleep quality	PSQI composite score	3.5	2.1

*SOL, sleep onset latency; TST, total sleep time; REM, rapid eye movement sleep; SE, sleep efficiency; TTL, total training load; pp, per participant; PSQI, Pittsburgh Sleep Quality Inventory.*

### Associations Between Sleep, Mental Strain, and Training Load

#### Same-Day Effects of Mental Strain and Training Load on Sleep

**FIGURE 4:**
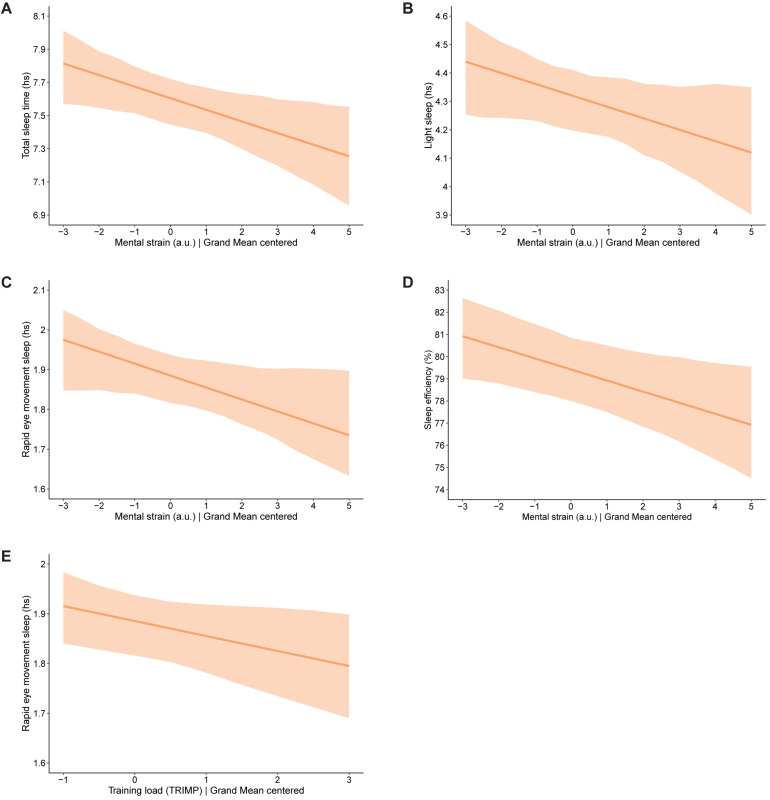
Significant findings identified when testing the same-day effects of mental strain and training load on sleep in 56 junior endurance athletes: **(A)** effect of mental strain on total sleep time, **(B)** effect of mental strain on light sleep, **(C)** effect of mental strain on rapid eye movement sleep, **(D)** effect of mental strain on sleep efficiency, and **(E)** effect of training load on rapid eye movement sleep. The bold line represents the average intercept and slope identified in the given models. The shaded area above the line represents the upper 2.5% of 95% credibility interval, while the shaded area below the line represents the lower 2.5% of 95% credibility interval. Value 0 on the *x*-axis refers to the average value on the measured outcome parameter.

Table 4 shows the between-level variation in the relationships between mental strain and training load (IVs) and sleep variables (DV) analyzed with random intercept and random slope models. In the table, TTL values have been scaled down by 100 in order to communicate the effects more clearly. The results show that, with each point increase on the mental strain score, time asleep, light sleep, REM sleep, and sleep efficiency decreased significantly. Moreover, with each point increase in TRIMP score, REM sleep decreased significantly. The significant findings are illustrated in Figure 4. There were significant variances in the strength of all tested relationships between athletes. In other words, both the intercepts and the effect of mental strain and training load on sleep were stronger in some athletes than others. The explained variance of the DVs by these models is low and ranges from 1.8 to 2.4%.

**TABLE 4:** Two-level random intercept and random slope models investigating the effect of mental strain and training load (IVs) on sleep variables (DVs) and whether these effects vary between individuals in 56 junior endurance athletes.

		Means					Variances					
			
DV	Coefficients	Est.	P. SD	Lower 2.5% of 95% CI	Upper 2.5% of 95% CI	Sig.	Est.	P. SD	Lower 2.5% of 95% CI	Upper 2.5% of 95% CI	Sig.	*R*^2^
Sleep onset latency (h)	Intercept	0.56	0.03	0.49	0.62	< 0.001*	0.05	0.01	0.03	0.07	< 0.001*	2.0%
	Slope * MS	0.00	0.01	–0.02	0.01	0.480	0.00	0.00	0.00	0.00	< 0.001*	
	Slope * TTL	–0.01	0.02	–0.03	0.02	0.360	0.00	0.00	0.00	0.01	< 0.001*	
Total sleep time (h)	Intercept	7.61	0.06	7.49	7.73	< 0.001*	0.18	0.05	0.12	0.32	< 0.001*	1.8%
	Slope * MS	–0.07	0.02	–0.12	–0.03	< 0.001*	0.01	0.01	0.00	0.02	< 0.001*	
	Slope * TTL	–0.02	0.03	–0.09	0.04	0.280	0.01	0.01	0.00	0.03	< 0.001*	
Light sleep (h)	Intercept	4.32	0.05	4.24	4.41	< 0.001*	0.11	0.03	0.07	0.19	< 0.001*	1.9%
	Slope * MS	–0.04	0.02	–0.08	–0.00	*0*.005*	0.01	0.00	0.00	0.02	< 0.001*	
	Slope * TTL	0.02	0.02	–0.03	0.05	0.245	0.00	0.00	0.00	0.01	< 0.001*	
Deep sleep (h)	Intercept	1.40	0.02	1.36	1.44	< 0.001*	0.03	0.01	0.02	0.05	< 0.001*	2.4%
	Slope * MS	–0.01	0.01	–0.03	0.01	0.120	0.00	0.00	0.00	0.00	< 0.001*	
	Slope * TTL	0.00	0.01	–0.02	0.03	0.420	0.00	0.00	0.00	0.01	< 0.001*	
REM sleep (h)	Intercept	1.89	0.03	1.84	1.94	< 0.001*	0.04	0.01	0.02	0.06	< 0.001*	2.0%
	Slope * MS	–0.03	0.01	–0.05	–0.00	*0*.005*	0.00	0.00	0.00	0.00	< 0.001*	
	Slope * TTL	–0.03	0.02	–0.06	–0.00	*0*.015*	0.00	0.00	0.00	0.01	< 0.001*	
Sleep efficiency (%)	Intercept	79.53	0.70	78.22	80.94	< 0.001*	5.26	6.21	16.76	42.10	< 0.001*	1.8%
	Slope * MS	–0.50	0.22	–0.95	–0.09	*0*.005*	0.67	0.55	0.08	2.04	< 0.001*	
	Slope * TTL	0.08	0.24	–0.46	0.46	0.330	0.36	0.36	0.04	1.50	< 0.001*	

Values refer to the between-level. Significant results are based on Bayesian estimation, and are marked with an *. R^2^ values refer to within-level explained variance averaged across athletes. Abbreviations: P. SD, Posterior standard deviation; CI, credibility interval; DV, dependent variable; ND, next day; TRIMP, training impulse; au, arbitrary units; SOL, sleep onset latency; TST, total sleep time; LS, light sleep; SWS, deep sleep; REM, rapid eye movement sleep; SE, sleep efficiency.

#### Next Day Effects of Sleep on Mental Strain and Training Load

**FIGURE 5:**
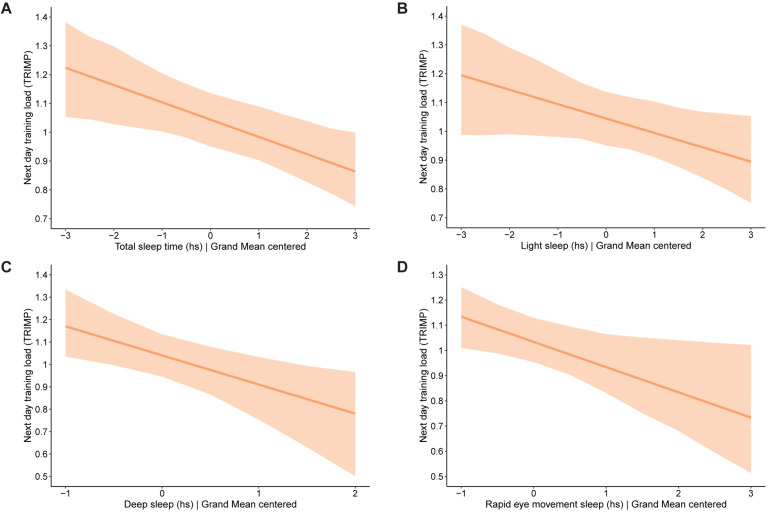
Significant findings identified when testing the effects of sleep on mental strain and training load the next day in 56 junior endurance athletes: **(A)** effect of total sleep time on next day training load, **(B)** effect of light sleep on next day training load, **(C)** effect of deep sleep on next day training load, and **(D)** effect of rapid eye movement sleep on next day training load. The bold line represents the average intercept and slope identified in the given models. The shaded area above the line represents the upper 2.5% of 95% credibility interval, while the shaded area below the line represents the lower 2.5% of 95% credibility interval. Value 0 on the *x*-axis refers to the average value on the measured outcome parameter.

Table 5 shows the between-level variation in the relationships between sleep (IVs) and next day mental strain and training load (DV). In the table, TTL values have been scaled down by 100 in order to communicate the effects more clearly. The results show that with each hour increase on the sleep variables time asleep, light sleep, deep sleep and REM sleep, TRIMP score the next day decreased significantly (see Figure 5 for a visual representation of the significant findings). None of the sleep variables influenced mental strain the next day. There were significant variances in the strength of all tested relationships between athletes. In other words, both the intercepts, and the effect of sleep on next day mental strain and training load differed between athletes. The explained variance of the DVs by these models is low and ranges from 0.3 to 4.4%.

**TABLE 5:** Two-level random intercept and random slope models investigating the effect of sleep variables (IVs) on mental strain and training load (DVs) and whether these effects vary between individuals in 56 junior endurance athletes.

		Means					Variances					
			
DV	Coefficients	Est.	P. SD	Lower 2.5% of 95% CI	Upper 2.5% of 95% CI	Sig.	Est.	P. SD	Lower 2.5% of 95% CI	Upper 2.5% of 95% CI	Sig.	*R*^2^
ND mental strain (au)	Intercept	3.55	0.16	3.24	3.87	< 0.001*	1.45	0.32	1.04	2.24	< 0.001*	0.3%
	Slope * SOL	–0.02	0.07	–0.17	0.11	0.407	0.01	0.02	0.00	0.07	< 0.001*	
ND training load (TRIMP)	Intercept	1.04	0.04	0.95	1.12	< 0.001*	0.08	0.02	0.05	0.13	< 0.001*	0.4%
	Slope * SOL	0.01	0.04	–0.08	0.09	0.408	0.01	0.01	0.00	0.05	< 0.001*	
ND mental strain (au)	Intercept	3.57	0.17	3.25	3.88	< 0.001*	1.49	0.30	1.04	2.14	< 0.001*	1.9%
	Slope * TST	0.01	0.03	–0.06	0.05	0.425	0.02	0.01	0.01	0.04	< 0.001*	
ND training load (TRIMP)	Intercept	1.04	0.05	0.95	1.13	< 0.001*	0.08	0.02	0.05	0.13	< 0.001*	1.3%
	Slope * TST	–0.06	0.02	–0.09	–0.03	< 0.001*	0.00	0.00	0.00	0.01	< 0.001*	
ND mental strain (au)	Intercept	3.56	0.17	3.24	3.88	< 0.001*	1.48	0.29	1.04	2.13	< 0.001*	2.4%
	Slope * LS	0.00	0.04	–0.10	0.07	0.500	0.05	0.02	0.03	0.10	< 0.001*	
ND training load (TRIMP)	Intercept	1.04	0.05	0.95	1.13	< 0.001*	0.09	0.02	0.05	0.13	< 0.001*	0.9%
	Slope * LS	–0.05	0.02	–0.10	–0.01	*0*.010*	0.01	0.01	0.00	0.02	< 0.001*	
ND mental strain (au)	Intercept	3.55	0.16	3.26	3.89	< 0.001*	1.45	0.32	0.94	2.31	< 0.001*	1.7%
	Slope * SWS	0.06	0.09	–0.10	0.27	0.250	0.19	0.08	0.09	0.41	< 0.001*	
ND training load (TRIMP)	Intercept	1.04	0.04	0.95	1.12	< 0.001*	0.08	0.02	0.05	0.15	< 0.001*	1.3%
	Slope * SWS	–0.13	0.05	–0.22	–0.02	*0*.020*	0.03	0.02	0.01	0.09	< 0.001*	
ND mental strain (au)	Intercept	3.57	0.16	3.25	3.89	< 0.001*	1.47	0.29	1.03	2.13	< 0.001*	1.2%
	Slope * REM	–0.02	0.06	–0.14	0.06	0.365	0.07	0.04	0.02	0.16	< 0.001*	
ND training load (TRIMP)	Intercept	1.03	0.05	0.94	1.12	< 0.001*	0.08	0.02	0.05	0.13	< 0.001*	1.3%
	Slope * REM	–0.10	0.04	–0.18	–0.03	*0*.010*	0.03	0.02	0.01	0.08	< 0.001*	
ND mental strain (au)	Intercept	3.54	0.16	3.25	3.89	< 0.001*	1.49	0.33	0.97	2.38	< 0.001*	3.4%
	Slope * SE	–0.00	0.01	–0.01	0.01	0.260	0.00	0.00	0.00	0.00	< 0.001*	
ND training load (TRIMP)	Intercept	1.04	0.04	0.94	1.11	< 0.001*	0.08	0.02	0.05	0.13	< 0.001*	4.4%
	Slope * SE	0.01	0.00	–0.00	0.02	0.050	0.00	0.00	0.00	0.00	< 0.001*	

Values refer to the between level. Significant results are based on Bayesian estimation, and are marked with an *. R^2^ values refer to within-level explained variance averaged across athletes. P. SD, Posterior standard deviation; CI, redibility interval; DV, dependent variable; ND, next day; TRIMP, training impulse; au, arbitrary units; SOL, sleep onset latency; TST, total sleep time; LS, light sleep; SWS, deep sleep; REM, rapid eye movement sleep; SE, sleep efficiency.

### The Effects of Subjective Sleep Quality

In all, 9 (16%) athletes were poor sleepers, and the rest (*N* = 47) were good sleepers. Four different types of cross-interaction analyses, described in section “Statistical Analyses,” investigated whether the strength of the relations explored in the previous step varied between individuals, and whether type of sleeper (good vs. poor) had an impact on the strength of those relations. Results of the analyses are presented in Table 6.

**TABLE 6:** Cross-interaction analyses investigating the moderating effects of subjective sleep quality on the effect of explanatory variables on dependent variables (see Figure 3 for overview of tested associations).

DV	Coefficients	Est.	P. SD	Lower 2.5% of 95% CI	Upper 2.5% of 95% CI	Sig.	*R*^2^
Next day mental strain	Intercept good sleeper	3.49	0.17	3.17	3.82	< 0.001*	
	Δ Intercept poor sleeper	0.45	0.47	–0.49	1.31	0.188	
	Good sleeper * SOL (h)	–0.08	0.05	–0.17	0.01	0.043	
	Δ Poor sleeper * SOL (h)	0.28	0.04	0.09	0.46	< 0.001*	39.6%
Next day training load	Intercept good sleeper	105.61	4.86	96.08	115.93	< 0.001*	
	Δ Intercept poor sleeper	–12.43	12.26	–36.15	11.71	0.157	
	Good sleeper * deep sleep (h)	–8.39	5.62	–18.92	2.25	0.063	
	Δ Poor sleeper * deep sleep (h)	–38.75	14.39	–67.65	–11.43	*0*.002*	21.9%

The interaction coefficients refer to the moderating effects of subjective sleep quality on the effect of the given explanatory and dependent variables. Values refer to the between level. Significant results are based on Bayesian estimation and are marked with an *. Δ represents the difference to the good sleeper’s value. Significant results are italicized. DV, dependent variable; P. SD, Posterior standard deviation; CI, credibility interval; MS, mental strain; REM, rapid eye movement sleep; SOL, sleep onset latency.

**FIGURE 6:**
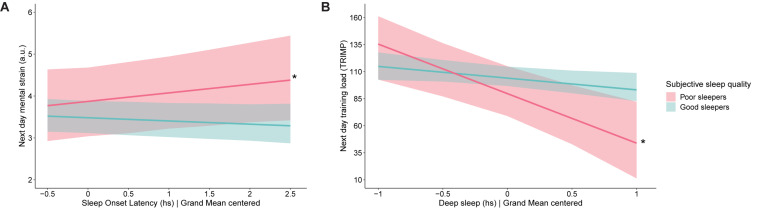
Significant findings identified when testing the moderating role of subjective sleep quality in 56 junior endurance athletes: **(A)** effect of sleep onset latency on next day mental strain and **(B)** effect of deep sleep on next day training load. The red bold line represents the average intercept and slope identified for poor sleepers, while the turquoise line represents the average intercept and slope identified for good sleepers. The shaded area above each line represents the upper 2.5% of 95% credibility interval, while the shaded area below each line represents the lower 2.5% of 95% credibility interval. Value 0 on the *x*-axis refers to the average value on the measured outcome parameter. * denotes significant difference between poor and good sleepers, *p* < 0.05.

The cross-level interaction analyses reveal fully moderating effects of subjective sleep quality on the associations between average SOL and next-day mental strain and average deep sleep and next-day training load. For poor sleepers, the strength of the associations are significantly different from that of good sleepers: with each hour of SOL, poor sleepers’ next-day mental strain increases by 0.20 points (as compared to −0.08 points for good sleepers), and with each hour of deep sleep, poor sleepers’ next-day training load decreases by 47.14 TRIMP points (as compared to 8.4 points for good sleepers). See Figure 6 for a visual representation of these findings. When predicting next-day mental strain by SOL and PSQI, the model shows that these variables explain 39.6% of the variance in next-day mental strain, and the model predicting next-day training load by PSQI and deep sleep explains 21.9% of the variance in next-day training load.

## Discussion

The present study recorded daily repeated measurements of sleep, mental strain, and training load over a period of 61 consecutive days in 56 junior endurance athletes in order to investigate reciprocal associations between sleep, mental strain, and training load as well as the moderating effects of being a good versus a poor sleeper. The main findings are (1) increases in mental strain are associated with decreased TST, REM sleep, and SE, and higher training load is associated with decreased REM sleep; (2) increases in TST, light, deep, and REM sleep are associated with decreased training load the next day; (3) in poor sleepers, increases in SOL are associated with increases in next-day mental strain, and increases in deep sleep are associated with decreases in next-day training load.

### Reciprocal Associations Between Sleep, Mental Strain, and Training Load

Increases in mental strain are associated with decreased TST and SE. These findings are in line with previous research, which elucidates the detrimental effect of mental strain on sleep. For example, experimental and observational data show that psychosocial (Kim and Dimsdale, 2007) and emotional (Vandekerckhove et al., 2011) stressors as well as worries and ruminations (Akerstedt et al., 2007; Van Laethem et al., 2016) and high trait-like vulnerability to stress-related sleep disturbance in non-insomniac individuals (Drake et al., 2004) are associated with decreased SE. Likewise, participants exposed to emotional stressors (Vandekerckhove et al., 2011) exhibit decreased TST. The present study extends these findings to the athletic population. On days with increased mental strain, the reductions in TST may be attributed to delayed bedtimes and the reductions in SE to more frequent awakenings. The feasibility of experimentally establishing causality between mental strain and sleep in athletes remains uncertain. Such studies are nevertheless greatly beneficial for the development of suitable interventions aimed at resolving the negative effects of mental strain on sleep in athletes exposed to mentally straining environments.

Increases in mental strain are also associated with decreased light and REM sleep. These results are striking as REM sleep is thought to play an important role in emotion regulation and processing (Palmer and Alfano, 2017), and existing literature shows increases in REM sleep in subjects with mood disorders (Armitage, 2007). These effects may be attributed to processing of negatively charged emotional and cognitive stimuli; if the daytime regulatory processes fail to fully resolve or integrate the stimuli, the processes may continue throughout the night, increasing REM sleep. However, decreases in REM in association with mentally straining stimuli are shown in healthy populations (Kim and Dimsdale, 2007). This may point to a different processing strategy as REM sleep may decrease as a result of a mentally straining stimulus and may only be compensated for after the stimulus is resolved and distress is normalized (Vandekerckhove et al., 2011). Experimental studies should establish whether REM sleep occurs later following the exposure to the mentally straining stimulus when the intensity of the stimulus has waned.

REM sleep is further reduced in association with increases in training load. There is some limited support for these findings, showing decreases in REM sleep as a result of acute exercise (Youngstedt et al., 1997) or increased training load (Kubitz et al., 1996; Brand et al., 2010). In the latter studies, decreases in REM are accompanied by concurrent increases in deep sleep, which may be indicative of an inhibitory influence of deep sleep on REM sleep. Such effects are not seen in the present study, possibly due to the circadian regulation of sleep (Youngstedt et al., 1997). A reduction in REM could be explained by late-evening exercise, which may reduce REM sleep if TST is not upheld. Indeed, disruptive effects of late-night exercise on sleep are well established (Thun et al., 2015). Unfortunately, we were unable to investigate the effects of timing of exercise; thus, future studies should consider the effects of exercise timing to elucidate potential associations with sleep.

Moreover, increases in TST, light, deep, and REM sleep are associated with decreases in training load the next day, highlighting how daily fluctuations in sleep parameters influence training load. Sleep-deprived elite athletes are previously shown to decrease their training load (Cook et al., 2012), which is a conceivable consequence of sleep loss, assumingly mediated by changes in motivation. In the present study, however, athletes were obtaining adequate sleep durations, which may have influenced the subsequent training load differently. We hypothesize that the increases in TST and sleep stages may reflect an increased need for recovery, which may have subsequently led to downregulation of training load. Such downregulation of training load may not be a detrimental aspect in athletes’ training progressions. In fact, reductions of training loads in response to increased recovery needs may reflect well-developed self-regulatory skills. These findings should be replicated in other studies. In addition, studies should investigate the associations between sleep debt accumulated over specific time periods with changes in training load to investigate how the need for recovery influences the chosen training load.

### The Moderating Role of Subjective Sleep Quality

In poor sleepers, increases in SOL are associated with increases in subsequent mental strain. Increased SOL, frequently attributed to mental activity at bedtime (e.g., worrying) (Wuyts et al., 2012), is common among poor sleepers (Paceschott et al., 1994). We hypothesize that, in the present sample, worrying at bedtime may have prolonged SOL. Upon awakening and if last night’s worries remained unresolved, athletes will conceivably continue worrying and subsequently report increased mental strain. If such interactions between sleep and mental strain continue, a disrupting cycle of worrying at bedtime and poor sleep may develop (Riemann et al., 2010). Indeed, associations between lower quality or quantity of sleep and subsequent increases in emotional and cognitive aspects of stress in healthy populations are previously demonstrated (Galambos et al., 2009). It is possible that such processes are responsible for the present associations in poor sleepers between increased SOL and increased next-day mental strain.

Finally, in poor sleepers, increases in deep sleep are associated with subsequent decreases in training load. This supplements the present associations between increased TST and the sleep stages and decreased training load. We, therefore, continue to hypothesize that increases in deep sleep may represent an increased need for recovery, which subsequently leads to downregulation of training load. Considering the roles of deep sleep in physiological recovery (Vyazovskiy and Delogu, 2014), these results may have consequences for athletic performance development and functioning. However, empirical studies fail to demonstrate a consistent relationship between these variables, showing increases in deep sleep (Shapiro et al., 1981) and also no effects (Knufinke et al., 2018; O’Donnell et al., 2019) following increases in physical load. It is possible that the present results bridge the gap in existing research by showing that the associations between deep sleep and training load only exist in poor sleepers. If that is the case, these results hold important practical implications for coaches and athlete support staff as athletes who struggle with sleep disturbances may be at higher risk of experiencing poor recovery or generally are in need for more or longer recovery than athletes who sleep well.

### Limitations

The results of the present study should be interpreted with several limitations in mind. First, while retaining the question structure, we adapted the questions from the original WQ (McLean et al., 2010) to fit the purpose of this study. Moreover, despite its frequent use for the monitoring of athletes’ well-being, the validity and reliability of the WQ have not been formally assessed. Further, the levels of mental strain in the current study are relatively low (mean 3.5 ± SD 1.6). The reasons for the mild mental strain may be due to the high prevalence of good sleepers or that athletes generally cope well with their situation. Therefore, the results based on the mental strain scale should be interpreted with these limitations in mind. Second, we only find a small proportion of explained variance in the analysis of reciprocal associations between sleep, mental strain, and training load (aim 1). This is likely due to the fact that a plethora of different variables may have influenced the associations at play. Such variables may include coping styles, personality traits, regularity of sleep/wake patterns, chronotype, or relationship with coaches. In the present study, these variables are unaccounted for. In addition, the current sample includes only 9 poor sleepers, and the rest of the sample are good sleepers. Therefore, the statistical analyses exploring whether the reciprocal associations between sleep, mental strain, and training load are moderated by sleeper category (good vs. poor) should be interpreted with caution. Last, although the device used for sleep monitoring used in the current study performed well in the validation study against PSG, the relationship of the Somnofy-derived sleep parameters and PSG data is not perfect (Toften et al., 2020). Hence, some measurement error regarding the sleep variables should be acknowledged.

## Conclusion

The present study provides novel results on how variations in mental strain and training load influence sleep patterns in junior endurance athletes and vice versa. Mental strain is inversely associated with subsequent TST, REM sleep, and SE, and training load is inversely associated with subsequent REM sleep. As many competitive athletes face mentally and physically straining sport-related situations, these results hold potentially important implications for informing the future development of specific sleep and mental health–related interventions for this group. Further, we show that increased sleep durations and increased sleep stages (light, deep, and REM) are associated with subsequent decrease in athletes’ training load. These findings indicate an increased need for recovery and subsequent self-regulatory reduction of training loads by the athletes. Currently, there is consensus regarding neither how much sleep competitive athletes should obtain nor their optimal sleep stage distribution. Specifying competitive athletes’ individual sleep needs is crucial for optimization of athletes’ recovery. Last, in athletes subjectively categorized as poor sleepers, increases in SOL are associated with increases in subsequent mental strain. In the same group, increases in deep sleep are associated with decreases in subsequent training load. The present study is the first to identify the moderating effects of subjective sleep quality, which are especially relevant for the practice field. We suggest that coaches should pay close attention to athletes’ subjective experience of sleep as this person-level characteristic may be key in identifying athletes at risk for poor recovery.

## Data Availability Statement

The raw data supporting the conclusions of this article will be made available by the authors, without undue reservation, to any qualified researcher.

## Ethics Statement

The studies involving human participants were reviewed and approved by the Regional Committee for Medical and Health Research Ethics in Central Norway. Written informed consent from the participants’ legal guardian/next of kin was not required to participate in this study in accordance with the national legislation and the institutional requirements.

## Author Contributions

MH and FM designed and conceptualized the study. ØS and SP contributed in this process. FM facilitated contact with the schools involved in this study. MH collected the data and wrote the manuscript. FM and ØS supervised the data collection. CK supervised the statistical approach. MH and CK carried out the analyses. All authors were involved in the interpretation of the statistical analyses, editing of the manuscript, and approved the final version of the manuscript.

## Conflict of Interest

The authors declare that the research was conducted in the absence of any commercial or financial relationships that could be construed as a potential conflict of interest.

## Footnotes

1 https://olt-dagbok.nif.no/

2 https://info.bestr.no/

## References

[B1] Akerstedt T. Kecklund G. Axelsson J. (2007). Impaired sleep after bedtime stress and worries. *Biol. Psychol.* 76 170–173. 10.1016/j.biopsycho.2007.07.010 17884278

[B2] American Academy of Sleep Medicine (2020). *AASM Manual for the Scoring of Sleep and Associated Events.* Darien, IL: American Academy of Sleep Medicine.

[B3] Andrade A. Bevilacqua G. Casagrande P. Brandt R. Coimbra D. (2019). Sleep quality associated with mood in elite athletes. *Phys. Sportsmed.* 47 312–317. 10.1080/00913847.2018.1553467 30477376

[B4] Armitage R. (2007). Sleep and circadian rhythms in mood disorders. *Acta Psychiatr. Scand.* 115 104–115. 10.1111/j.1600-0447.2007.00968.x 17280576

[B5] Bender A. Van Dongen H. Samuels C. (2018). Sleep quality and chronotype differences between elite athletes and non-athlete controls. *Clocks Sleep* 1 3–12. 10.3390/clockssleep1010002 33089151 PMC7509668

[B6] Boulay M. R. Simoneau J. A. Lortie G. Bouchard C. (1997). Monitoring high-intensity endurance exercise with heart rate and thresholds. *Med. Sci. Sports Exerc.* 29 125–132. 10.1097/00005768-199701000-00018 9000165

[B7] Brand S. Gerber M. Beck J. Hatzinger M. Puhse U. Holsboer-Trachsler E. (2010). Exercising, sleep-EEG patterns, and psychological functioning are related among adolescents. *World J. Biol. Psychiatry* 11 129–140. 10.3109/15622970903522501 20109114

[B8] Buchheit M. Racinais S. Bilsborough J. C. Bourdon P. C. Voss S. C. Hocking J. (2013a). Monitoring fitness, fatigue and running performance during a pre-season training camp in elite football players. *J. Sci. Med. Sport* 16 550–555. 10.1016/j.jsams.2012.12.003 23332540

[B9] Buchheit M. Simpson B. M. Garvican-Lewis L. A. Hammond K. Kley M. Schmidt W. F. (2013b). Wellness, fatigue and physical performance acclimatisation to a 2-week soccer camp at 3600 m (ISA3600). *Br. J. Sports Med.* 47(Suppl. 1), i100–i106. 10.1136/bjsports-2013-092749 24282195 PMC3903314

[B10] Buysse D. J. Reynolds C. F. III Monk T. H. Berman S. R. Kupfer D. J. (1989). The pittsburgh sleep quality index: a new instrument for psychiatric practice and research. *Psychiatry Res.* 28 193–213. 10.1016/0165-1781(89)90047-4 2748771

[B11] Campbell E. Irving R. Bailey J. Dilworth L. A. Abel W. (2018). Overview of psychophysiological stress and the implications for junior athletes. *Am. J. Sports Sci. Med.* 6 72–78.

[B12] Conte D. Kolb N. Scanlan A. T. Santolamazza F. (2018). Monitoring training load and well-being during the in-season phase in national collegiate athletic association division i men’s basketball. *Int. J. Sports Physiol. Perform.* 13 1067–1074. 10.1123/ijspp.2017-0689 29431544

[B13] Cook C. Beaven C. M. Kilduff L. P. Drawer S. (2012). Acute caffeine ingestion’s increase of voluntarily chosen resistance-training load after limited sleep. *Int. J. Sport Nutr. Exerc. Metab.* 22 157–164. 10.1123/ijsnem.22.3.157 22349085

[B14] Coughlin S. S. (1990). Recall bias in epidemiologic studies. *J. Clin. Epidemiol.* 43 87–91. 10.1016/0895-4356(90)90060-3 2319285

[B15] Drake C. Richardson G. Roehrs T. Scofield H. Roth T. (2004). Vulnerability to stress-related sleep disturbance and hyperarousal. *Sleep* 27 285–291. 10.1093/sleep/27.2.285 15124724

[B16] Foster C. (1998). Monitoring training in athletes with reference to overtraining syndrome. *Med. Sci. Sports Exerc.* 30 1164–1168. 10.1097/00005768-199807000-00023 9662690

[B17] Galambos N. L. Dalton A. L. Maggs J. L. (2009). Losing sleep over it: daily variation in sleep quantity and quality in canadian students’. First Semester of University. *J. Res. Adoles.* 19 741–761. 10.1111/j.1532-7795.2009.00618.x

[B18] Gallo T. F. Cormack S. J. Gabbett T. J. Lorenzen C. H. (2016). Pre-training perceived wellness impacts training output in Australian football players. *J. Sports Sci.* 34 1445–1451. 10.1080/02640414.2015.1119295 26637525

[B19] Grimm P. (2010). “Social desirability bias,” in *Wiley International Encyclopedia of Marketing*, eds Sheth J. Malhotra N. (Hoboken, NJ: Wiley).

[B20] Hanton S. Fletcher D. Coughlan G. (2005). Stress in elite sport performers: a comparative study of competitive and organizational stressors. *J. Sports Sci.* 23 1129–1141. 10.1080/02640410500131480 16194989

[B21] Hooper S. L. Mackinnon L. T. (1995). Monitoring overtraining in athletes - recommendations. *Sports Med.* 20 321–327. 10.2165/00007256-199520050-00003 8571005

[B22] Hrozanova M. Moen F. Pallesen S. (2019). Unique predictors of sleep quality in junior athletes: the protective function of mental resilience, and the detrimental impact of sex, worry and perceived stress. *Front. Psychol.* 10:1256. 10.3389/fpsyg.2019.01256 31214076 PMC6554288

[B23] Juliff L. E. Halson S. L. Peiffer J. J. (2015). Understanding sleep disturbance in athletes prior to important competitions. *J. Sci. Med. Sport* 18 13–18. 10.1016/j.jsams.2014.02.007 24629327

[B24] Jung C. M. Melanson E. L. Frydendall E. J. Perreault L. Eckel R. H. Wright K. P. (2011). Energy expenditure during sleep, sleep deprivation and sleep following sleep deprivation in adult humans. *J. Physiol. Lon.* 589 235–244. 10.1113/jphysiol.2010.197517 21059762 PMC3039272

[B25] Kellmann M. Bertollo M. Bosquet L. Brink M. Coutts A. J. Duffield R. (2018). Recovery and performance in sport: consensus statement. *Int. J. Sports Physiol. Perform,.* 13 240–245. 10.1123/ijspp.2017-0759 29345524

[B26] Kim E. J. Dimsdale J. E. (2007). The effect of psychosocial stress on sleep: a review of polysomnographic evidence. *Behav. Sleep Med.* 5 256–278. 10.1080/15402000701557383 17937582 PMC4266573

[B27] Knufinke M. Nieuwenhuys A. Geurts S. A. E. Most E. I. S. Maase K. Moen M. H. (2018). Train hard, sleep well? Perceived training load, sleep quantity and sleep stage distribution in elite level athletes. *J. Sci. Med. Sport* 21 427–432. 10.1016/j.jsams.2017.07.003 28754605

[B28] Kolling S. Steinacker J. M. Endler S. Ferrauti A. Meyer T. Kellmann M. (2016). The longer the better: sleep-wake patterns during preparation of the world rowing junior championships. *Chronobiol. Int.* 33 73–84. 10.3109/07420528.2015.1118384 26730643

[B29] Kroshus E. Wagner J. Wyrick D. Athey A. Bell L. Benjamin H. J. (2019). Wake up call for collegiate athlete sleep: narrative review and consensus recommendations from the NCAA Interassociation Task Force on Sleep and Wellness. *Br. J. Sports Med.* 53 731–736. 10.1136/bjsports-2019-100590 31097460

[B30] Kubitz K. A. Landers D. M. Petruzzello S. J. Han M. (1996). The effects of acute and chronic exercise on sleep. A meta-analytic review. *Sports Med.* 21 277–291. 10.2165/00007256-199621040-00004 8726346

[B31] Lastella M. Lovell G. P. Sargent C. (2014). Athletes’ precompetitive sleep behaviour and its relationship with subsequent precompetitive mood and performance. *Eur. J. Sport Sci.* 14(Suppl. 1), S123–S130. 10.1080/17461391.2012.660505 24444196

[B32] Lastella M. Roach G. D. Vincent G. E. Scanlan A. T. Halson S. L. Sargent C. (2020). The impact of training load on sleep during a 14-day training camp in elite, adolescent, female basketball players. *Int. J. Sports Physiol. Perform.* 15 724–730. 10.1123/ijspp.2019-0157 32015213

[B33] Leeder J. Glaister M. Pizzoferro K. Dawson J. Pedlar C. (2012). Sleep duration and quality in elite athletes measured using wristwatch actigraphy. *J. Sports Sci.* 30 541–545. 10.1080/02640414.2012.660188 22329779

[B34] Lukonaitiene I. Kamandulis S. Paulauskas H. Domeika A. Pliauga V. Kreivyte R. (2020). Investigating the workload, readiness and physical performance changes during intensified 3-week preparation periods in female national Under18 and Under20 basketball teams. *J. Sports Sci.* 38 1018–1025. 10.1080/02640414.2020.1738702 32164498

[B35] Mah C. D. Sparks A. J. Samaan M. A. Souza R. B. Luke A. (2019). Sleep restriction impairs maximal jump performance and joint coordination in elite athletes. *J. Sports Sci.* 37 1981–1988. 10.1080/02640414.2019.1612504 31122131 PMC8889916

[B36] Manzar M. D. Zannat W. Hussain M. E. (2015). Sleep and physiological systems: a functional perspective. *Biol. Rhythm Res.* 46 195–206. 10.1080/09291016.2014.966504

[B37] Marino M. Li Y. Rueschman M. N. Winkelman J. W. Ellenbogen J. M. Solet J. M. (2013). Measuring sleep: accuracy, sensitivity, and specificity of wrist actigraphy compared to polysomnography. *Sleep* 36 1747–1755. 10.5665/sleep.3142 24179309 PMC3792393

[B38] McLean B. D. Coutts A. J. Kelly V. McGuigan M. R. Cormack S. J. (2010). Neuromuscular, endocrine, and perceptual fatigue responses during different length between-match microcycles in professional rugby league players. *Int. J. Sports Physiol. Perform.* 5 367–383. 10.1123/ijspp.5.3.367 20861526

[B39] Muthén B. (2010). *Bayesian Analysis in Mplus: A Brief Introduction.* Princeton, NJ: Citeseer.

[B40] Muthén L. Muthén B. (2017). *Mplus User’s Guide*, 8th Edn Los Angeles, CA: Muthén and Muthén.

[B41] Nixdorf I. Frank R. Beckmann J. (2015). An explorative study on major stressors and its connection to depression and chronic stress among German elite athletes. *Adv. Phys. Educ.* 5 255–262. 10.4236/ape.2015.54030

[B42] O’Donnell S. L. Beaven C. M. Jacobson G. M. Bird S. Driller M. W. (2019). Melatonin and sleep responses following exercise in elite female athletes. *Sport Exerc. Sci. N. Z.* 3, 23–33.

[B43] O’Donnell S. Beaven C. M. Driller M. W. (2018). From pillow to podium: a review on understanding sleep for elite athletes. *Nat. Sci. Sleep* 10 243–253. 10.2147/NSS.S158598 30197545 PMC6112797

[B44] Paceschott E. F. Kaji J. Stickgold R. Hobson J. A. (1994). Nightcap measurement of sleep quality in self-described good and poor sleepers. *Sleep* 17 688–692. 10.1093/sleep/17.8.688 7701179

[B45] Pallesen S. Grønli J. Myhre K. Moen F. Bjorvatn B. Hanssen I. (2018). A pilot study of impulse radio ultra wideband radar technology as a new tool for sleep assessment. *J. Clin. Sleep Med.* 14 1249–1254. 10.5664/jcsm.7236 29991417 PMC6040792

[B46] Pallesen S. Nordhus I. H. Omvik S. Sivertsen B. Matthiesen S. B. Bjorvatn B. (2005). Pittsburgh sleep quality index. *Tidsskrift-Norsk Psykologforening* 42:714.

[B47] Palmer C. A. Alfano C. A. (2017). Sleep and emotion regulation: an organizing, integrative review. *Sleep Med. Rev.* 31 6–16. 10.1016/j.smrv.2015.12.006 26899742

[B48] Peake J. M. Kerr G. Sullivan J. P. (2018). A critical review of consumer wearables, mobile applications, and equipment for providing biofeedback, monitoring stress, and sleep in physically active populations. *Front. Physiol.* 9:743. 10.3389/fphys.2018.00743 30002629 PMC6031746

[B49] Podsakoff P. M. MacKenzie S. B. Lee J. Y. Podsakoff N. P. (2003). Common method biases in behavioral research: a critical review of the literature and recommended remedies. *J. Appl. Psychol.* 88 879–903. 10.1037/0021-9010.88.5.879 14516251

[B50] Pyne D. B. Martin D. T. (2011). “Fatigue-Insights from individual and team sports,” in *Regulation of Fatigue in Exercise*, ed. Marino F. E. (New York, NY: Nova Science), 177–185.

[B51] Riemann D. Spiegelhalder K. Feige B. Voderholzer U. Berger M. Perlis M. (2010). The hyperarousal model of insomnia: a review of the concept and its evidence. *Sleep Med. Rev.* 14 19–31. 10.1016/j.smrv.2009.04.002 19481481

[B52] Sargent C. Halson S. Roach G. D. (2014). Sleep or swim? Early-morning training severely restricts the amount of sleep obtained by elite swimmers. *Eur. J. Sport Sci.* 14(Suppl. 1), S310–S315. 10.1080/17461391.2012.696711 24444223

[B53] Seiler K. S. Kjerland G. O. (2006). Quantifying training intensity distribution in elite endurance athletes: is there evidence for an “optimal” distribution? *Scand. J. Med. Sci. Sports* 16 49–56. 10.1111/j.1600-0838.2004.00418.x 16430681

[B54] Shapiro C. M. Bortz R. Mitchell D. Bartel P. Jooste P. (1981). Slow-wave sleep: a recovery period after exercise. *Science* 214 1253–1254. 7302594 10.1126/science.7302594

[B55] Solli G. S. Tonnessen E. Sandbakk O. (2017). The training characteristics of the world’s most successful female cross-country skier. *Front. Physiol.* 8:1069. 10.3389/fphys.2017.01069 29326603 PMC5741652

[B56] Solli G. S. Tonnessen E. Sandbakk O. (2019). Block vs. traditional periodization of HIT: two different paths to success for the world’s best cross-country skier. *Front. Physiol.* 10:375. 10.3389/fphys.2019.00375 31024338 PMC6460991

[B57] Sylta O. Tonnessen E. Seiler S. (2014). Do elite endurance athletes report their training accurately? *Int. J. Sports Physiol. Perform.* 9 85–92. 10.1123/Ijspp.2013-0203 23921186

[B58] Teng E. Lastella M. Roach G. Sargent C. (2011). “The effect of training load on sleep quality and sleep perception in elite male cyclists,” in *Little Clock, Big Clock: Molecular to Physiological Clocks*, eds Kennedy G. Sargent C. (Melbourne: Australasian Chronobiology Society), 5–10.

[B59] Thun E. Bjorvatn B. Flo E. Harris A. Pallesen S. (2015). Sleep, circadian rhythms, and athletic performance. *Sleep Med. Rev.* 23 1–9. 10.1016/j.smrv.2014.11.003 25645125

[B60] Toften S. Pallesen S. Hrozanova M. Moen F. Grønli J. (2020). Validation of sleep stage classification using non-contact radar technology and machine learning (Somnofy§). *Sleep Med*. 75 54–61. 10.1016/j.sleep.2020.02.022 32853919

[B61] Van Laethem M. Beckers D. G. J. van Hooff M. L. M. Dijksterhuis A. Geurts S. A. E. (2016). Day-to-day relations between stress and sleep and the mediating role of perseverative cognition. *Sleep Med.* 23 71–79. 10.1016/j.sleep.2016.06.020 27810189

[B62] Vandekerckhove M. Weiss R. Schotte C. Exadaktylos V. Haex B. Verbraecken J. (2011). The role of presleep negative emotion in sleep physiology. *Psychophysiology* 48 1738–1744. 10.1111/j.1469-8986.2011.01281.x 21895689

[B63] Vyazovskiy V. V. Delogu A. (2014). NREM and REM sleep: complementary roles in recovery after wakefulness. *Neuroscientist* 20 203–219. 10.1177/1073858413518152 24598308

[B64] Walker M. P. Stickgold R. (2004). Sleep-dependent learning and memory consolidation. *Neuron* 44 121–133. 10.1016/j.neuron.2004.08.031 15450165

[B65] Wuyts J. De Valck E. Vandekerckhove M. Pattyn N. Bulckaert A. Berckmans D. (2012). The influence of pre-sleep cognitive arousal on sleep onset processes. *Int. J. Psychophysiol.* 83 8–15. 10.1016/j.ijpsycho.2011.09.016 21963535

[B66] Youngstedt S. D. O’Connor P. J. Dishman R. K. (1997). The effects of acute exercise on sleep: a quantitative synthesis. *Sleep* 20 203–214. 10.1093/sleep/20.3.203 9178916

